# An audit of CT brain findings in adults with new-onset seizures in a resource restricted setting in South Africa

**DOI:** 10.4102/sajr.v26i1.2294

**Published:** 2022-01-20

**Authors:** Sabelo H. Mabaso, Deepa Bhana-Nathoo, Susan Lucas

**Affiliations:** 1 Department of Radiology, Faculty of Health Sciences, Chris Hani Baragwanath Hospital, University of the Witwatersrand, Johannesburg, South Africa

**Keywords:** new-onset, first-onset, adult-onset, seizure, CT findings

## Abstract

**Background:**

Globally, adults presenting with seizures account for 1% – 2% of visits to emergency departments (EDs), of which 25% are new-onset seizures. Neuroimaging is essential as part of the initial workup. Multiple studies have demonstrated abnormal CT brain (CTB) findings in these patients.

**Objectives:**

To review the CTB findings in adults presenting with new-onset seizures in a resource restricted setting.

**Method:**

A retrospective review of 531 CTBs was conducted at a tertiary hospital in Gauteng on adults presenting to the ED with new-onset seizures.

**Results:**

The mean age of the patients was 45.6 ± 17.1 years, and the male to female ratio was 1.2:1. Generalised and focal seizure types were almost equally represented. Of the total 531 patients, 168 (31.6%) were HIV positive. The CTB findings were abnormal in 257 (48.4%) patients, albeit vascular pathology accounted for 21.9%. Infective pathology accounted for 14.1% with a statistically significant association with HIV (*p* = 0.003). Trauma related pathology was 2.4%, whilst neoplastic pathology was seen in 3.0%. Other causes included congenital pathology, calcifications, atrophy and gliosis. Clinical factors associated with abnormal CTB findings were age ≥ 40 years, HIV infection, hypertension, focal seizures, low Glasgow Coma Scale (GCS), raised cerebrospinal fluid (CSF) protein and presence of lymphocytes.

**Conclusion:**

A high yield of abnormal CTB findings was noted in adult patients who presented with new-onset seizures, supporting the use of urgent CTB in patients with certain clinical risk factors. Patients without these risk factors can be scanned within 24–48 h in a resource restricted setting.

## Introduction

Globally, adults presenting with seizures account for up to 2% of visits to medical emergency departments (EDs)^1,2,3,4^ and of these, 25% are new-onset seizures.^1,5^ It is estimated that 5% – 10% of the population will experience a seizure in their lifetime.^3,6,7^

According to Gavvala and Schuele,^5^ seizures represent ‘a transient occurrence of signs and symptoms due to excessive neuronal activity in the brain’. They may be classified simply as focal (affecting a single cerebral hemisphere), generalised (affecting both hemispheres) or unknown^1,5^ In a clinical setting, seizures are defined in a specific manner. Gavvala and Scheule^5^ described them as follows: unprovoked seizure, occurring without precipitating factors; acute asymptomatic seizure, occurring in close temporal relationship with a transient brain insult; focal seizure, affecting one part of cerebral hemisphere; generalised seizure, distributed in both cerebral hemispheres; and epilepsy, an enduring predisposition to generate seizures.

The estimated incidence rate of adult-onset seizures is 81.7 per 100 000 per year in developing countries compared to 45.0 per 100 000 per year in developed countries, as reported by Ba-Diop et al.^8^ The incidence rate of new-onset adult seizures is higher in HIV positive populations.^9^ Another study noted that the majority of patients that were severely immunosuppressed had an underlying, identifiable intracranial abnormality,^10^ confirming the predisposition of patients with advanced HIV to opportunistic infections as a cause of new-onset seizures.

Patients with new-onset seizures require imaging as part of their clinical workup.^1^ Multiple international studies have demonstrated varying degrees of abnormal CT brain (CTB) findings,^3,5,7^ however, the urgency or optimal timing of imaging has not yet been determined. Computed tomography is the initial imaging of choice, being the most practical, especially in the emergency setting and for those requiring follow-up imaging to monitor disease progression.^6,7^ Magnetic resonance imaging (MRI) may be utilised as supplementary imaging, where CTB findings are inconclusive or for further characterisation.^3,5^

The purpose of this study was to determine the underlying CTB findings in adults with new-onset seizures and to identify specific risk factors, if any, that may contribute to the urgency or timing of imaging.

## Research methods and design

A retrospective cross-sectional study of 531 adult patients was conducted, reviewing the CTB findings in cases with new-onset seizures at a tertiary hospital in Gauteng, South Africa, between 01 January 2016 and 31 December 2018. Patients with missing data, illegible reports, those presenting with acute trauma, repeat scans for patients with a known diagnosis and CTB findings not reviewed by a consultant radiologist were excluded from the study.

Clinical information including patient age, sex, type of seizure (generalised, focal or status epilepticus) and co-morbidities (including HIV status) were accessed and recorded from the hospital picture archiving and communication system (PACS). Relevant laboratory results were accessed from the National Health Laboratory Services (NHLS) and relevant clinical data were recorded and tabulated using an anonymous data collection sheet.

Categorical variables were expressed as numbers and percentages and compared with the chi-square test. Continuous variables with a normal distribution were expressed as means and standard deviation (s.d.). The median and interquartile ranges (IQR) were used for continuous variables with a non-normal distribution. The one-way analysis of variance (ANOVA) was used to compare normally distributed continuous variables and the Kruskal-Wallis rank test was used to compare medians for non-normal data. Differences between groups were considered statistically significant at *p* < 0.05. All analyses were conducted using STATA MP version 13.0 (StataCorp, Texas).

### Ethical considerations

The study was approved by Human Research Ethics Committee of the University of the Witwatersrand, certificate number M190609. Participant consent was not required as this was a retrospective record review and to maintain strict anonymity, no personally identifiable information was recorded.

## Results

Of the included 531 patients, 285 (53.7%) were male. The ages ranged from 18 to 95 years with a mean age of 45.6 years (s.d.: ± 17.1) and a median age of 44 years (IQR: 32–59 years). Demographic data and major abnormal findings are presented in Table 1.

**TABLE 1:** Demographic data of patients presenting with new-onset seizures.

Characteristics	All patients (*n* = 531)	HIV positive (*n* = 168)	Abnormal CTB (*n* = 257)	Major categories of abnormal CTB	
				Vascular (*n* = 116)	Infective (*n* = 75)
%	100.0	31.6	48.4	21.9	14.1
Mean age (years)	45.6	42.4	51.3	59.6	41.0
Median age (years)	44.0	41.0	50.0	62.0	40.0
M: F (male: female)	1.2: 1	1: 1.2	1.4: 1	1: 1.1	1.8: 1

CTB, CT brain; HIV, human immunodeficiency virus.

### Clinical and laboratory data

There were 94 (17.7%) focal seizures, 95 (17.9%) generalised seizures and 14 (2.6%) cases of status epilepticus; 328 (61.8%) were undocumented. The Glascow Coma Scale (GCS) was documented in 104 patients with a median GCS of 13 (IQR: 9–14). The GCS was < 8 in 16 (15.4%) patients, between 9 and 12 in 29 (27.9%) patients and between 13 and 15 in 59 (56.7%) patients. Abnormal CTB findings were more frequent in patients with a GCS *≤* 12 (*p* = 0.175).

A total of 168 (31.6%) patients were HIV positive, while 225 (42.4%) had undocumented HIV statuses. There was a significantly high prevalence of HIV in the 30–49 years age group (*p* < 0.001). The CD4 count was documented in 124 HIV positive patients with a median CD4 count of 229 cells/μL (IQR: 52 cells/μL – 430 cells/μL); 48.4% were below 200 cells/μL.

Hypertension was present in 78 (14.7%) patients. There was a statistically significant prevalence of hypertension above the age of 49 years (*p* < 0.001). Diabetes mellitus was documented in 23 (4.3%) patients and was significantly prevalent above the age of 59 years (*p* = 0.001). There were 16 (3.0%) patients with chronic kidney disease and 42 (7.9%) with other co-morbidities including 18 (3.3%) cases of tuberculosis, 11 (2.1%) cases of known primary malignancy, 4 (0. 8%) cases of eclampsia, 6 (1.1%) cases of systemic lupus erythematosus and 3 (0.6%) cases of liver disease.

Laboratory data included renal function, full blood count and cerebrospinal fluid (CSF) analysis. A high prevalence of abnormal CTB findings was seen in patients with a white cell count above 10.0 × 10^9^/L (*p* = 0.011). Abnormal CTB findings were also more prevalent in patients with a CSF protein above 0.45 g/L (*p* = 0.033); CSF lymphocytosis was significantly associated with abnormal CTB findings (*p* = 0.004).

### CT brain findings

**FIGURE 1:**
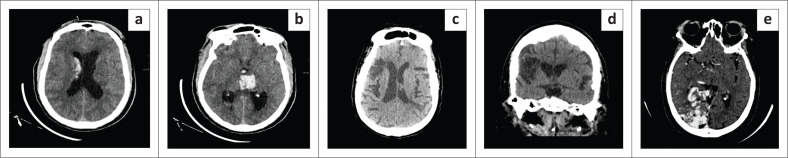
Vascular pathology as a cause of adult-onset seizures. (a and b) Hypertensive intraparenchymal haemorrhage in a 56-year-old male with newly diagnosed hypertension. Axial non-enhanced CT (NECT): Left thalamic haemorrhage with minimal surrounding oedema. Associated intraventricular blood. (c and d) Chronic right middle cerebral artery territory infarction in an adult patient with index episode of seizures. Axial and coronal NECT: Right MCA territory wedge-shaped hypodensity with mild right lateral ventricular ex vacuo dilatation. (e) Right occipital lobe arteriovenous malformation in a 53-year-old female with no background medical history. Axial contrast enhanced CT (CECT): ‘Bag of worms’ appearance of draining and feeding vessels in the right occipital lobe.

The CTB findings were abnormal in 48.4% of the cases. The aetiologies of the abnormal CTB findings are presented in Table 2. Of the 116 vascular cases, 60 (51.7%) had infarcts, 12 (10.3%) intracerebral haemorrhages and 3 (2.6%) vascular malformations. There were 41 (35.4%) cases of vascular pathologies related to white matter changes, of which most were suggestive of chronic small vessel ischemic disease and posterior reversible encephalopathy syndrome. Selected images of vascular related pathology are demonstrated in Figure 1.

**TABLE 2:** Proportions of abnormal CTB findings and cause categories in patients with new-onset seizures.

Neuroimaging findings	Frequency (*n* = 531)	Percentage
**CTB findings:**		
Normal CTB findings	274	51.6
**Abnormal CTB findings**	**257**	**48.4**
**Pathology categories:**		
Vascular	116	21.9
Infective	75	14.1
Neoplastic	16	3.0
Trauma	13	2.4
Congenital	5	0.9
**Other**	**32**	**6.0**

CTB, CT brain.

**FIGURE 2:**
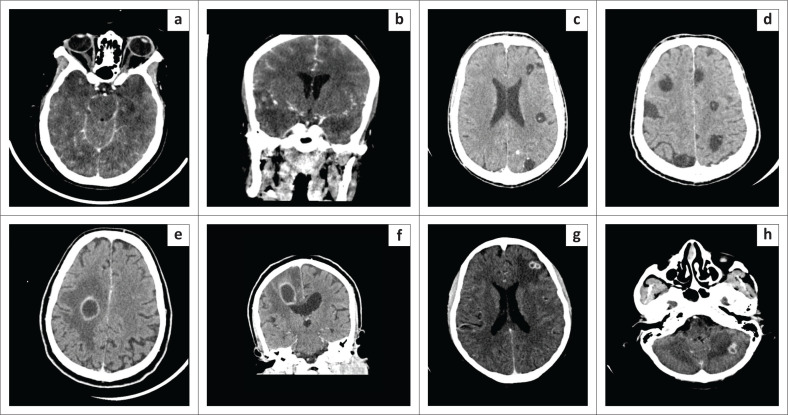
Infective pathology as a cause of adult-onset seizures. (a and b) Herpes Simplex (HSV) encephalitis in a young immunocompromised female patient. Axial NECT: Bilateral anterior temporal lobe oedema with hyperdense foci in keeping with microhaemorrhages. (c and d) Neurocysticercosis in a 47-year-old male with newly diagnosed HIV. Axial - contrast enhanced CT (CECT): Multiple bilateral non-enhancing round CSF density cystic intra-axial lesions of varying sizes with central hyperdense scolices (dot sign). (e and f) HIV positive patient on tuberculosis treatment. Coronal and axial CECT: Large right frontal lobe ring-enhancing lesion adjacent to the right lateral ventricle. Surrounding vasogenic oedema with associated mass effect. A differential diagnosis of a pyogenic or a tuberculous abscess was given. (g and h) A 53-year-old HIV positive male patient. CD4 = 26 cells/μL. Axial CECT: Multiple ring-enhancing lesions with vasogenic oedema in the cerebellum and corticomedullary junction of cerebrum. A differential of toxoplasmosis and tuberculomas was given.

Seven (9.3%) of the 75 infective cases had features suggestive of meningitis, 6 (8.0%) pyogenic abscesses, 14 (18.7%) neurocysticercosis and 3 (4.0%) toxoplasmosis. The other cases were attributable to tuberculosis, cryptococcosis and miscellaneous infective white matter disease disorders. Of the tuberculosis cases, 7 (9.3%) had tuberculous meningitis, whilst 12 (16.0%) were attributable to tuberculomas and tuberculous abscesses. There were 26 (34.7%) cases of white matter changes related to infection, of which herpes encephalitis was seen in 6 (8.0%) patients, cerebritis in 5 (6.7%) cases, suspected HIV encephalopathy in 9 (12%) cases and progressive multifocal leukoencephalopathy in 6 (8.0%) cases. Selected images of infective pathology are shown in Figure 2.

**FIGURE 3:**
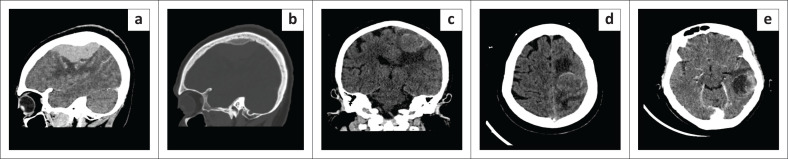
Neoplastic pathology as a cause of adult-onset seizures. (a and b) Meningioma in a 64-year-old female with worsening of left arm weakness and new-onset seizures. Sagittal contrast enhanced CT (CECT). (brain and bone window): Large right frontal avidly enhancing extra-axial mass with associated hyperostosis of the adjacent skull inner table as well as mass effect and oedema of the adjacent parenchyma. (c and d) Left frontal lobe mass in a 59-year-old female who presented with new-onset seizures. Axial and coronal non-contrast CT (NCCT): Large left frontal lobe solid round intra-axial heterogeneously hyperdense mass with surrounding vasogenic oedema and mass effect on the frontal horn of the left lateral ventricle. A differential diagnosis of metastases or lymphoma was given. e: Metastasis from endometroid adenocarcinoma of the endometrium. 69-year-old female with endometroid adenocarcinoma of the endometrium who presented with first onset seizures. Axial CECT: Large ring-enhancing solid-cystic intra-axial mass in the left temporal lobe corticomedullary junction with surrounding oedema.

Neoplastic pathologies were seen in 16 (3.0%) of the patients, of which 9 were primary central nervous system (CNS) neoplasms whilst 7 were secondary CNS neoplasms. Selected images of neoplastic pathology are shown in Figure 3. The CT findings in neoplastic or infective cases were diagnosed largely on clinical and imaging work up and treated accordingly. Not all cases had histological or microbiological confirmation at the time of the study.

**FIGURE 4:**
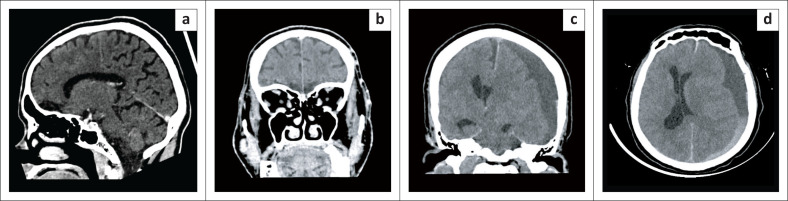
Trauma related pathology as a cause of adult-onset seizures. (a and b) Bilateral rectus gyri gliosis in a 49-year-old male with new-onset seizures. Sagittal and coronal contrast enhanced CT (CECT): Hypodensities of bilateral rectus gyri consistent with gliosis. (c and d) Left acute subdural hematoma in a 72-year-old male with new-onset seizures and confusion. Axial and coronal non-enhanced CT (NECT): Large left mixed density subdural hematoma with a haematocrit level. Associated subfalcine and uncal herniation with right lateral ventricle entrapment hydrocephalus.

A total of 13 (2.4%) traumatic pathologies were recorded. These were subdural hematoma and old depressed fractures with underlying encephalomalacia. Selected images of trauma related pathology are shown in Figure 4.

Of the 5 (0.9%) cases with congenital pathology, frontoethmoidal encephalocele accounted for 2 cases, with the other 3 cases being arachnoid cyst, schizencephaly and Dandy-Walker malformation. A total of 32 (6.0%) unclassified abnormal CTB findings were recorded, 5 cases of calcifications, 5 of atrophy and 22 of gliosis.

### Associations with various CTB abnormalities

Cerebral oedema was present in 40 cases, midline shift in 26 cases, hydrocephalus in 32 cases and herniation in 10 cases. Extra-axial collections were seen in 8 cases associated with infective pathologies. Venous sinus thrombosis was noted in 2 cases associated with infection. Infarction was present in 12 cases associated with infective pathology. There was higher parietal lobe involvement with vascular and infective pathologies. The basal ganglia were more involved with vascular pathology, whilst extra-axial involvement was seen more in infective and traumatic pathologies. This data is presented in Table 3.

**TABLE 3:** CTB abnormality in patients with new-onset seizures characterised by site of involvement.

Pathology of abnormal CTB	Site of involvement (*n* = 531)													
	Frontal lobe		Parietal lobe		Temporal lobe		Occipital lobe		Cerebellum and brainstem		Basal ganglia		Extra-axial	
	*n*	%	*n*	%	*n*	%	*n*	%	*n*	%	*n*	%	*n*	%
Vascular	56	10.6	70	13.2	53	10.0	33	6.2	3	0.6	45	8.5	0	0
Infective	34	6.4	42	7.0	19	3.6	17	3.2	11	2.1	10	1.9	8	1.5
Neoplastic	5	0.9	4	0.8	5	0.9	4	0.8	1	0.2	1	0.2	4	0.8
Trauma	11	2.1	8	1.5	6	1.1	1	0.2	1	0.2	1	0.2	10	1.9

CTB, CT brain.

The mean age for vascular pathology was 59.6 years and for infective causes, 41.0 years (Table 1). Patients with focal seizures had a high prevalence of abnormal CTB findings (*p* = 0.560). Of the patients with neoplastic pathologies that had a recorded seizure type, 50% had focal seizures. The relationship between various pathology categories and seizure type is shown in Table 4.

**TABLE 4:** Relationship between seizure type and abnormal CTB categories.

Seizure type	Causes of abnormal CTB (*n* = 531)											
	Vascular		Infective		Neoplastic		Trauma		Congenital		Other	
	*n*	%	*n*	%	*n*	%	*n*	%	*n*	%	*n*	%
Focal	28	5.3	12	2.3	8	1.5	3	0.6	2	0.4	4	0.8
Generalised	18	3.4	21	4.0	2	0.4	4	0.8	1	0.2	3	0.6
Status epilepticus	3	0.6	2	0.4	0	0	1	0.2	1	0.2	0	0
Undocumented	67	12.6	40	7.5	6	1.1	5	0.9	1	0.2	25	5.7

CTB, CT brain.

A large proportion of HIV positive patients had a higher prevalence of infective causes on abnormal CTB imaging (*p* = 0.003). There were more infarcts, intracerebral haemorrhages, chronic small vessel ischemic white matter disease and posterior reversible encephalopathy syndrome in patients with hypertension.

## Discussion

Relating to demographics, this study demonstrated a gender distribution that was similar to other studies with a male to female ratio of 1.2:1. A study by Zarmehri et al. also consisted of more males, accounting for 62% of their study population.^11^ In contrast, in a retrospective study performed in South Africa by Smith et al, their study sample consisted of more females, accounting for 61.2%.^7^ Another study by Kaur et al. had 65% male subjects.^12^

The mean age for abnormal CT brain findings in this study was 51.3 years. In the study by Smith et al.,^7^ the common age of presentation of new-onset seizures was between 31 and 40 years. In another study by Zarmehri et al., their study population had a mean age of 39.78 ± 17 years.^11^ Additionally, this study revealed that elderly patients had more abnormal CTB findings. More vascular pathologies were seen in older patients and more infective pathologies in younger and HIV infected patients. Kaur et al. showed that 68.1% of patients aged below 40 years had idiopathic seizures.^12^ This observation suggests that older patients require CTB imaging more urgently than HIV negative younger patients.

Clinically, Smith et al. found that generalised seizures were the commonest seizure type, representing 86.7% of their patients.^7^ A high prevalence of generalised seizures were also reported by Kaur et al. at 59%.^12^ In contrast, this study showed that focal and generalised seizures were almost equally represented when the seizure type was documented. There was a high prevalence of abnormal CTB scans in patients with focal seizures, but this was not statistically significant (*p* = 0.560). Yang et al. demonstrated similar findings in children, but no comparable study was found in adults.^13^

The GCS value was recorded in 104 (19.6%) patients. Although there was a high number of abnormal CTB scans seen in patients with GCS ≤ 12 (29/45 [64.4%]), this number was not statistically significant (*p* = 0.175). Despite the lack of statistical significance, this observation is similar to international literature. Wang et al. demonstrated that altered level of consciousness is a strong predictor of abnormal CTB findings.^14^ In another study by Moolla et al. that looked at adult-onset seizures in HIV positive patients, GCS less than 15 was found to predict space occupying lesions and cerebral oedema.^9^ The lack of statistical significance in this study may be attributable to the low proportion of recorded levels of consciousness.

The HIV prevalence of 31.6% in this study was higher than the 2018 Statistics South Africa report, which estimated the prevalence of HIV at 13.1%. The age group commonly affected by HIV in our study was similar to the 2018 Statistics South Africa report which states that 15–49-year-old patients are predominantly affected.^15^ There was a statistically significant relationship between HIV and infective pathology accounting for abnormal CTB findings (*p* = 0.003), owing to vulnerability to opportunistic intracranial infections in this group. In a study by Sinha et al., the majority of HIV infected patients had opportunistic central nervous system infections, ranging from tuberculosis, cryptococcosis, toxoplasmosis and HIV encephalitis.^16^ The infections demonstrated in this study included pyogenic abscesses, neurocysticercosis, toxoplasmosis, tuberculosis and cryptococcosis.

The other common comorbidities included diabetes mellitus, hypertension and chronic kidney disease, all of which were common in older patients. However, only hypertension showed a statistically significant relationship with abnormal CTB findings (*p* = 0.012), including infarcts, intracerebral haemorrhages, chronic small vessel ischemic white matter disease and posterior encephalopathy syndrome. This could be explained by an increased risk of stroke and other intracranial complications associated with hypertension. Hesdorffer et al.^17^ found that severe uncontrolled hypertension increased the risk of unprovoked seizures and they postulated the mechanism to be that of epileptogenic white matter changes caused by hypertension. Hypertension related central nervous system complications such as intracerebral haemorrhage, subarachnoid haemorrhage and posterior reversible syndrome increase the likelihood of abnormal CTB findings.^18^

There was a significant association between a high white cell count (above 10.0 × 10^9^/L) and abnormal CTB findings (*p* = 0.011). This may be related to infective pathologies but we found no supporting literature in this regard. A study by Khalili et al. found that a raised white cell count has a low positive predictive value and low specificity for bacterial meningitis.^19^ Elevated CSF protein (above 0.45 g/L) was significantly associated with abnormal CTB findings (*p* = 0.033) in this study, although this is a retrospective association since lumbar puncture is usually performed after CTB imaging. Zisimopoulou et al. found a correlation between abnormal CSF protein and unprovoked first episodes of seizure, thought to be related to disruption of the blood brain barrier.^20^ No studies have looked at the direct correlation between abnormal CSF protein and CTB findings. The presence of high CSF lymphocytes showed a statistically significant correlation with abnormal CTB findings (*p* = 0.004). This is supported by the findings in the study by Zisimopoulou et al., where a high CSF cell count correlated with abnormal CTB findings in 71 patients.^20^

There were 48.4% abnormal CTB findings in this study. According to the literature, abnormal CTB findings range from 10% to 68.9%,^5,6,21^ most likely underlined by the demographic differences in patients studied as suggested by Adams et al.^2^ Additionally, this study did not differentiate between CTBs performed with or without intravenous iodinated contrast as no significant impact was anticipated. Studied literature indicated that administration of intravenous contrast does little to change the sensitivity of CTB findings.^1,22,23,24^

Vascular pathology accounted for 21.9% of abnormal CTB findings. These results are similar to a South African study by Smith et al., where vascular pathology contributed a larger proportion of CTB abnormalities, with infarcts accounting for 25% and intracerebral haemorrhage 12.5%.^7^ Similarly, Kaur et al. showed that stroke was the most common cause of abnormal CTB findings in India, accounting for 23%.^12^ Vascular causes of abnormal CTB were more likely in the patients over 60 years and included ischemic infarction, chronic small vessel white matter disease and intracerebral haemorrhage. This finding confirms that age is a major risk factor for cerebral vascular disease. Yousufuddin and Young noted that ‘aging is the most robust non-modifiable risk factor for stroke, which doubles every 10 years after age 55 years’.^25^ Similarly, Kaur et al., demonstrated that 95.6% of seizures above the age of 40 years were secondary to stroke.^12^ This study also showed a high occurrence of vascular pathology in the parietal lobe, which is likely due to a higher incidence of stroke in the MCA distribution. This finding is supported by Ng et al., who found that MCA territory infarcts accounted for 50.8% of all ischemic strokes.^26^

Infective pathology constituted 14.1% of abnormal CTB findings and was seen in younger patients and the HIV infected. In the literature, the range of infective pathology ranged between 5.2% and 11.5%.^7,12^ There was a high frequency of infective pathology in the parietal lobe, likely due to a higher incidence of haematogenous sources of cerebral infection, which is also likely to involve the middle cerebral arterial territory. A review article by Patel et al. reported that hematogenous spread accounted for 9% – 43% of cerebral abscesses.^27^

Neoplastic pathology contributed 3.0% to the abnormal CTB findings, occurring in older patients with a mean age of 57.6 years. This is slightly different from the international literature, where neoplastic pathology contributed 8% – 14.8% of abnormal CTB findings.^11,12^ Focal seizures were seen in 50% of cases with neoplastic pathology but there was poor documentation of seizure type and statistical significance was not reached.

The fourth common cause of abnormal CTB finding was trauma related pathology, contributing 2.4%. These were largely subdural hematomas and old depressed fractures with underlying encephalomalacia. There was a high frequency of traumatic pathology in the frontal lobe. Frontal lobe pathology is more likely to present with seizures, behavioural and personality changes, whilst other areas of brain involvement will have additional focal neurological deficits.^28^

Congenital pathology as abnormal CTB finding in this study was an unexpected finding in patients over the age of 18 years who were unlikely to have their first seizure as adults. It is possible that patients may have been unconscious around the time of the seizure event and were unable to provide a history of previous seizures or there was a lack of thorough history taking. On the other hand, this may be due to loss to follow-up or caregivers seeking alternative opinion in a different health centre. The case of an arachnoid cyst in this study was likely an incidental finding and not the cause of the seizure, as arachnoid cysts are usually asymptomatic.^29^

Cerebral oedema, midline shift and hydrocephalus were the associated findings predominantly seen with infective and neoplastic pathologies. These findings guide the need for urgent medical or neurosurgical intervention. CTB is an ideal imaging modality for the emergent identification of these associated findings due to its ease of access and relatively low cost.^30^ This study did not investigate the follow-up management of the patients with these associated CTB findings.

### Study limitations

This retrospective study was limited by poor documentation of seizure type by the referring clinicians. As a result, *p*-values were calculated on smaller sample sizes and likely underestimate the power of our findings and the ability to investigate for predictors of abnormal CTB findings.

## Conclusion

A high yield of CTB imaging in adult new-onset seizures was demonstrated, with vascular and infective pathologies being the most common. Age ≥ 40 years, HIV infection, hypertension, focal seizures, low GCS, raised CSF protein and lymphocytes were identified as predictors of an abnormal CTB, and patients with these risk factors should be scanned urgently for prompt detection of medical and neurosurgical emergencies. Other patients without these risk factors, can be imaged as soon as possible within 24–48 h in resource restricted settings.
